# Healthy US population reference values for CT visceral fat measurements and the impact of IV contrast, HU range, and spinal levels

**DOI:** 10.1038/s41598-022-06232-5

**Published:** 2022-02-11

**Authors:** Brian A. Derstine, Sven A Holcombe, Brian E Ross, Nicholas C Wang, Stewart C Wang, Grace L Su

**Affiliations:** Michigan Medicine, Ann Arbor, MI USA

**Keywords:** Image processing, Obesity, Biomarkers

## Abstract

Measurements of visceral adipose tissue cross-sectional area and radiation attenuation from computed tomography (CT) scans provide useful information about risk and mortality. However, scan protocols vary, encompassing differing vertebra levels and utilizing differing phases of contrast enhancement. Furthermore, fat measurements have been extracted from CT using different Hounsfield Unit (HU) ranges. To our knowledge, there have been no large studies of healthy cohorts that reported reference values for visceral fat area and radiation attenuation at multiple vertebra levels, for different contrast phases, and using different fat HU ranges. Two-phase CT scans from 1,677 healthy, adult kidney donors (age 18–65) between 1999 and 2017, previously studied to determine healthy reference values for skeletal muscle measures, were utilized. Visceral adipose tissue cross-sectional area (VFA) and radiation attenuation (VFRA) measures were quantified using axial slices at T10 through L4 vertebra levels. T-tests were used to compare males and females, while paired t-tests were conducted to determine the effect (magnitude and direction) of (a) contrast enhancement and (b) different fat HU ranges on each fat measure at each vertebra level. We report the means, standard deviations, and effect sizes of contrast enhancement and fat HU range. Male and female VFA and VFRA were significantly different at all vertebra levels in both contrast and non-contrast scans. Peak VFA was observed at L4 in females and L2 in males, while peak VFRA was observed at L1 in both females and males. In general, non-contrast scans showed significantly greater VFA and VFRA compared to contrast scans. The average paired difference due to contrast ranged from 1.6 to − 8% (VFA) and 3.2 to − 3.0% (VFRA) of the non-contrast value. HU range showed much greater differences in VFA and VFRA than contrast. The average paired differences due to HU range ranged from − 5.3 to 22.2% (VFA) and − 5.9 to 13.6% (VFRA) in non-contrast scans, and − 4.4 to 20.2% (VFA) and − 4.1 to 12.6% (VFRA) in contrast scans. The − 190 to − 30 HU range showed the largest differences in both VFA (10.8% to 22.2%) and VFRA (7.6% to 13.6%) compared to the reference range (− 205 to − 51 HU). Incidentally, we found that differences in lung inflation result in very large differences in visceral fat measures, particularly in the thoracic region. We assessed the independent effects of contrast presence and fat HU ranges on visceral fat cross-sectional area and mean radiation attenuation, finding significant differences particularly between different fat HU ranges. These results demonstrate that CT measurements of visceral fat area and radiation attenuation are strongly dependent upon contrast presence, fat HU range, sex, breath cycle, and vertebra level of measurement. We quantified contrast and non-contrast reference values separately for males and females, using different fat HU ranges, for lumbar and thoracic CT visceral fat measures at multiple vertebra levels in a healthy adult US population.

## Introduction

The amount, distribution, and quality of fat in the core region of the body have significant clinical implications^1,2^. Visceral adiposity has been associated more accurately with cardiometabolic abnormalities than standard body measurements such as body mass index (BMI)^3^. Both the area and radiation attenuation of visceral fat have been associated with important clinical outcomes including mortality^2^. In the Framingham Heart Study, decreasing abdominal visceral fat attenuation was associated with worse cardiovascular outcome and greater cardiometabolic risk^2, 4^, while increased pericardial and intrathoracic fat was associated with decreased adiponectin and increased metabolic risk^5^. In patients with liver disease, higher visceral fat attenuation has been associated with higher mortality in patients with chronic liver disease and hepatocellular carcinoma^6–8^.

Visceral fat measurements can be inferred from methodologies such as anthropometry, dual energy X-ray absorptiometry (DXA), and bioelectrical impedance analysis (BIA). Higher-cost methods such as magnetic resonance (MR) and computed tomography (CT) can definitively delineate visceral from subcutaneous fat, however, MR is more expensive, slower, and less common than CT. Therefore, using existing CT scans acquired for clinical indications can be a convenient source of detailed body composition information without additional cost or risk of radiation. However, routine CTs have differential protocols, including differences in contrast administration, and different ranges of Hounsfield Unit (HU) values have been used to define fat pixels, both of which may affect fat measurements.

A few studies have investigated the effect of contrast phase on CT visceral fat measurement in clinical cohorts, generally reporting that VFA is higher in non-contrast compared contrast phase scans, though effect sizes varied widely and different fat HU ranges were used^9–14^.

The definition of visceral fat measured from CT depends upon the HU range used to define and extract fat pixels from imaging. Commonly used HU ranges include − 150 to − 50 HU^9–11^, − 195 to − 45 HU^15–17^, − 190 to − 30 HU^18–21^, and − 250 to − 50 HU^22^. We used − 205 to − 51 HU based on the adult fat threshold preset in Materialize Mimics software (version 17.0).

Studies recommend measuring visceral fat at the L4-L5 level to maximize correlation between axial fat areas and whole body fat volumes^18, 19, 21, 23^. However, the L4-L5 level is not available in all clinical CT scan protocols.

Previous work has reported healthy reference values for skeletal muscle measures using similarly large cohorts of kidney donor candidates^24–26^.

To our knowledge, no study has reported sex-specific, contrast and non-contrast reference values for visceral fat area and radiation attenuation in a large, healthy, US adult cohort, utilizing different fat HU ranges. The aim of this study is to quantify the effect of contrast and fat HU range on fat measurements and report reference values. Understanding these effects on visceral fat area and density would allow us to better leverage the important information that is present in CT scans performed for clinical indications.

## Results

### Cohort summary

The majority of subjects (1093, 65.2%) eventually donated a kidney while the remainder (584, 34.8%) had no record of donation. Comparing females to males (F/M), there was no significant difference in donor proportion (65.0/65.5%, *P* = 0.816) or race (62.4/62.0% Caucasian, 9.6/8.3% African American, 3.9/3.9% Other, *P* = 0.794) (Table 1). Females were older (41.9/39.65yr, *P* < 0.001), shorter (1.64/1.78 m, *P* < 0.001) and weighed less (72.4/88.0 kg, *P* < 0.001), and had slightly lower BMI (26.9/27.6 kg/m^2^, 2 = 0.003) compared to males.

**Table 1:** Cohort summary demographics split by sex.

	Female			Male			*P*
	n	Mean	SD	n	Mean	SD	
Age (yr)	1042	41.90	11.68	635	39.65	11.69	< **0.001**
Height (m)	1042	1.64	0.06	635	1.78	0.07	< **0.001**
Weight (kg)	1042	72.40	14.02	635	88.03	15.01	< **0.001**
**BMI (kg/m**^**2**^)	1042	26.94	4.90	635	27.60	4.11	**0.003**
Underweight	11	1.1%		0	0.0%		
Normal	394	37.8		168	26.5%		
Overweight	360	34.5%		318	50.1%		
Obese class I	207	19.9%		110	17.3%		
Obese class II	58	5.6%		37	5.8%		
Obese class III	12	1.2%		2	0.3%		
**Donor**							0.816
No	365	35.0%		219	34.5%		
Yes	677	65.0%		416	65.5%		
**Race**							0.794
African American	100	9.6%		53	8.3%		
Caucasian	650	62.4%		394	62.0%		
Not Reported	251	24.1%		163	25.7%		
Other	41	3.9%		25	3.9%		

Donor and race proportion *P*-value from Chi-squared test, all others from t-test comparing Females to Males.

### Reference values

Sex-specific mean and standard deviation (s.d.) healthy, adult reference values for VFA and VFRA at T10–L4 vertebra levels are reported separately for contrast and non-contrast phase for the reference − 205 to − 50 HU range (Table 2). Peak VFA (contrast/noncontrast) was observed at L4 in females (79.8/84.9 cm^2^) and L2 in males (141.4/148 cm^2^) while trough VFA was observed at T11 in females (51.6/53.9 cm^2^) and T10 in males (98.2/97.1 cm^2^). Peak VFRA was observed at L1 in both females (− 92.2/− 90.8HU) and males (− 95.5/− 94.3HU) while trough VFRA was observed at T10 in both females (− 97.0/− 98.1HU) and males (− 99.9/− 101.0HU).

**Table 2:** Male and Female mean and standard deviation for T10-L4 VFA and VFRA split by contrast status, vertebra level.

			Female			Male		
			n	Mean	SD	n	Mean	SD
VFA (cm^2^)	Contrast	T10	283	52.1	28.7	165	98.2	55.7
		T11	814	51.6	35.5	494	107.6	68.0
		T12	1024	58.6	44.1	625	123.9	79.6
		L1	1040	69.6	49.8	635	140.3	85.6
		L2	1036	74.3	50.5	633	141.4	85.3
		L3	1025	77.0	50.9	627	131.9	78.7
		L4	966	79.8	45.6	580	116.3	63.4
	Non-contrast	T10	283	55.6	28.8	165	97.1	52.6
		T11	814	53.9	35.5	494	109.6	69.0
		T12	1024	60.7	45.7	625	127.6	82.3
		L1	1040	71.7	52.1	635	145.0	89.1
		L2	1036	77.9	53.7	633	148.0	89.7
		L3	1025	81.9	53.7	627	141.1	82.5
		L4	966	84.9	48.3	580	123.5	66.3
VFRA (*HU*)	Contrast	T10	283	− 97.0	5.2	165	− 99.9	4.8
		T11	814	− 94.7	5.2	494	− 98.3	5.4
		T12	1024	− 92.4	5.9	625	− 96.2	5.8
		L1	1040	− 92.2	6.4	635	− 95.5	6.3
		L2	1036	− 93.4	6.4	633	− 96.6	6.7
		L3	1025	− 94.8	6.1	627	− 97.8	6.4
		L4	966	− 95.3	5.4	580	− 96.7	5.9
	Non-contrast	T10	283	− 98.1	5.2	165	− 101.0	4.5
		T11	814	− 96.0	5.9	494	− 98.8	5.2
		T12	1024	− 92.8	5.9	625	− 95.9	5.4
		L1	1040	− 90.8	6.2	635	− 94.3	6.1
		L2	1036	− 91.9	6.1	633	− 95.4	6.4
		L3	1025	− 93.5	5.8	627	− 96.5	6.4
		L4	966	− 93.8	5.1	580	− 95.2	5.8

All measures use the − 205 to − 51 HU reference range.

### Contrast versus non-contrast

VFA was greater in non-contrast versus contrast scans for all mean paired differences, except for those at T10 in males. In males at T10 the differences were not significantly different for all HU ranges. Across all vertebra levels and all HU ranges, mean paired differences ranged from − 3.0 to − 8.1% in women and 1.6 to − 8.0% in men, expressed as percent of the mean non-contrast VFA (Table 3).

**Table 3:** Mean VFA paired differences (contrast–non-contrast) raw ($\mu _d$) and as percent of overall mean non-contrast value ($\mu _{nc}$).

			Female					Male				
		HU range	n	$\mu _{nc}$	$\mu _d$	$\mu _d$/$\mu _{nc}$ (%)	*P*	n	$\mu _{nc}$	$\mu _d$	$\mu _d$/$\mu _{nc}$ (%)	*P*
VFA (cm^2^)	T10	− 150 to − 50 HU	283	52.7	− 2.8	− 5.6	< **0.001**	165	92.8	1.5	1.6	0.300
		− 190 to − 30 HU	283	67.5	− 4.9	− 7.8	< **0.001**	165	113.6	− 0.8	− 0.7	0.576
		− 195 to − 45 HU	283	58.6	− 3.7	− 6.7	< **0.001**	165	101.3	0.8	0.8	0.563
		− 205 to − 51 HU	283	55.6	− 3.5	− 6.7	< **0.001**	165	97.1	1.1	1.1	0.431
		− 250 to − 50 HU	283	58.4	− 4.4	− 8.1	< **0.001**	165	100.6	0.0	− 0.0	0.976
	T11	− 150 to − 50 HU	814	51.9	− 2.0	− 4.0	< **0.001**	494	106.3	− 2.2	− 2.1	**0.004**
		− 190 to − 30 HU	814	65.8	− 4.0	− 6.4	< **0.001**	494	126.9	− 4.3	− 3.5	< **0.001**
		− 195 to − 45 HU	814	57.0	− 2.6	− 4.8	< **0.001**	494	114.2	− 2.4	− 2.2	**0.002**
		− 205 to − 51 HU	814	53.9	− 2.4	− 4.6	< **0.001**	494	109.6	− 2.0	− 1.9	**0.008**
		− 250 to − 50 HU	814	56.0	− 3.0	− 5.8	< **0.001**	494	112.4	− 2.9	− 2.6	< **0.001**
	T12	− 150 to − 50 HU	1024	59.5	− 2.3	− 4.0	< **0.001**	625	125.4	− 4.7	− 3.9	< **0.001**
		− 190 to − 30 HU	1024	74.2	− 4.2	− 6.0	< **0.001**	625	147.3	− 6.5	− 4.6	< **0.001**
		− 195 to − 45 HU	1024	64.3	− 2.5	− 4.1	< **0.001**	625	133.0	− 4.3	− 3.3	< **0.001**
		− 205 to − 51 HU	1024	60.7	− 2.2	− 3.7	< **0.001**	625	127.6	− 3.8	− 3.0	< **0.001**
		− 250 to − 50 HU	1024	62.3	− 2.6	− 4.3	< **0.001**	625	129.9	− 4.3	− 3.4	< **0.001**
	L1	− 150 to − 50 HU	1040	70.6	− 2.5	− 3.7	< **0.001**	635	143.1	− 5.8	− 4.2	< **0.001**
		− 190 to − 30 HU	1040	87.3	− 4.7	− 5.6	< **0.001**	635	166.5	− 7.9	− 5.0	< **0.001**
		− 195 to − 45 HU	1040	75.9	− 2.6	− 3.6	< **0.001**	635	151.0	− 5.4	− 3.7	< **0.001**
		− 205 to − 51 HU	1040	71.7	− 2.1	− 3.0	< **0.001**	635	145.0	− 4.7	− 3.3	< **0.001**
		− 250 to − 50 HU	1040	73.2	− 2.4	− 3.4	< **0.001**	635	147.2	− 5.1	− 3.6	< **0.001**
	L2	− 150 to − 50 HU	1036	76.6	− 4.1	− 5.7	< **0.001**	633	145.9	− 7.8	− 5.6	< **0.001**
		− 190 to − 30 HU	1036	93.2	− 6.1	− 7.0	< **0.001**	633	167.4	− 9.5	− 6.0	< **0.001**
		− 195 to − 45 HU	1036	82.1	− 4.2	− 5.3	< **0.001**	633	153.4	− 7.2	− 4.9	< **0.001**
		− 205 to − 51 HU	1036	77.9	− 3.6	− 4.8	< **0.001**	633	148.0	− 6.6	− 4.6	< **0.001**
		− 250 to − 50 HU	1036	79.6	− 3.9	− 5.2	< **0.001**	633	150.0	− 7.0	− 4.9	< **0.001**
	L3	− 150 to − 50 HU	1025	80.5	− 5.5	− 7.3	< **0.001**	627	139.2	− 10.3	− 8.0	< **0.001**
		− 190 to − 30 HU	1025	95.9	− 7.0	− 7.9	< **0.001**	627	157.8	− 11.7	− 8.0	< **0.001**
		− 195 to − 45 HU	1025	85.7	− 5.4	− 6.7	< **0.001**	627	145.7	− 9.8	− 7.2	< **0.001**
		− 205 to − 51 HU	1025	81.9	− 4.9	− 6.4	< **0.001**	627	141.1	− 9.2	− 7.0	< **0.001**
		− 250 to − 50 HU	1025	83.5	− 5.2	− 6.7	< **0.001**	627	142.9	− 9.6	− 7.2	< **0.001**
	L4	− 150 to − 50 HU	966	83.5	− 5.7	− 7.3	< **0.001**	580	122.0	− 8.0	− 7.0	< **0.001**
		− 190 to − 30 HU	966	100.0	− 7.4	− 7.9	< **0.001**	580	140.8	− 9.8	− 7.5	< **0.001**
		− 195 to − 45 HU	966	89.0	− 5.6	− 6.7	< **0.001**	580	128.3	− 7.8	− 6.5	< **0.001**
		− 205 to − 51 HU	966	84.9	− 5.1	− 6.4	< **0.001**	580	123.5	− 7.2	− 6.2	< **0.001**
		− 250 to − 50 HU	966	86.5	− 5.4	− 6.7	< **0.001**	580	125.3	− 7.6	− 6.4	< **0.001**

*P*-values from paired t-test for T10–L4 with null hypothesis $\mu _d=0$. Negative values indicate contrast value lower than non− contrast value, on average. *P*-values <0.01 in bold.

The − 190 to − 30 HU range showed the largest paired differences in VFA between contrast and non-contrast scans, followed by − 150 to − 50 HU, − 250 to − 50 HU, − 195 to − 45 HU, and finally − 205 to − 51 HU. The largest differences between contrast and non-contrast were found at L3, L4, and T10 in females and at L3 and L4 in males, across all HU ranges.

VFRA was greater in non-contrast versus contrast scans for all mean paired differences between L1 and L4 in males and females, however, for T10–T12 the results differed by HU range and sex (Table 4).

**Table 4:** Mean VFRA paired differences (contrast–non-contrast) raw ($\mu _d$) and as percent of absolute mean non-contrast value ($\mu _d/|\mu _{nc}|$).

			Female					Male				
		HU range	n	$\mu _{nc}$	$\mu _d$	$\mu _d/|\mu _{nc}|$ (%)	*P*	n	$\mu _{nc}$	$\mu _d$	$\mu _d/|\mu _{nc}|$ (%)	*P*
VFRA (*HU*)	T10	− 150 to − 50 HU	283	− 90.7	− 0.1	− 0.1	0.551	165	− 94.7	− 0.1	− 0.1	0.600
		− 190 to − 30 HU	283	− 84.8	0.0	− 0.0	0.946	165	− 89.1	− 0.7	− 0.7	0.026
		− 195 to − 45 HU	283	− 93.6	0.6	0.7	0.014	165	− 97.1	0.4	0.4	0.236
		− 205 to − 51 HU	283	− 98.1	1.1	1.1	< **0.001**	165	− 101.0	1.0	1.0	**0.004**
		− 250 to − 50 HU	283	− 103.8	2.8	2.8	< **0.001**	165	− 105.5	2.8	2.7	< **0.001**
	T11	− 150 to − 50 HU	814	− 89.7	− 0.1	− 0.1	0.260	494	− 93.8	− 0.2	− 0.2	0.107
		− 190 to − 30 HU	814	− 82.9	− 0.2	− 0.2	0.289	494	− 88.0	− 0.8	− 0.9	< **0.001**
		− 195 to − 45 HU	814	− 91.7	0.7	0.8	< **0.001**	494	− 95.3	0.0	− 0.0	0.886
		− 205 to − 51 HU	814	− 96.0	1.3	1.4	< **0.001**	494	− 98.8	0.4	0.4	0.032
		− 250 to − 50 HU	814	− 100.8	3.1	3.2	< **0.001**	494	− 102.2	1.7	1.7	< **0.001**
	T12	− 150 to − 50 HU	1024	− 88.2	− 0.5	− 0.5	< **0.001**	625	− 92.3	− 0.4	− 0.5	< **0.001**
		− 190 to − 30 HU	1024	− 80.3	− 1.1	− 1.3	< **0.001**	625	− 85.9	− 1.3	− 1.5	< **0.001**
		− 195 to − 45 HU	1024	− 88.8	− 0.1	− 0.1	0.299	625	− 92.8	− 0.7	− 0.7	< **0.001**
		− 205 to − 51 HU	1024	− 92.8	0.3	0.4	0.018	625	− 95.9	− 0.4	− 0.4	0.016
		− 250 to − 50 HU	1024	− 95.8	1.6	1.7	< **0.001**	625	− 98.0	0.4	0.4	0.056
	L1	− 150 to − 50 HU	1040	− 87.3	− 1.4	− 1.6	< **0.001**	635	− 91.7	− 0.9	− 1.0	< **0.001**
		− 190 to − 30 HU	1040	− 79.2	− 2.4	− 3.0	< **0.001**	635	− 85.2	− 1.9	− 2.2	< **0.001**
		− 195 to − 45 HU	1040	− 87.2	− 1.7	− 1.9	< **0.001**	635	− 91.6	− 1.4	− 1.5	< **0.001**
		− 205 to − 51 HU	1040	− 90.8	− 1.4	− 1.5	< **0.001**	635	− 94.3	− 1.2	− 1.2	< **0.001**
		− 250 to − 50 HU	1040	− 92.8	− 0.7	− 0.7	< **0.001**	635	− 95.7	− 0.7	− 0.8	< **0.001**
	L2	− 150 to − 50 HU	1036	− 88.4	− 1.5	− 1.6	< **0.001**	633	− 92.8	− 0.8	− 0.9	< **0.001**
		− 190 to − 30 HU	1036	− 80.9	− 2.4	− 2.9	< **0.001**	633	− 86.9	− 1.9	− 2.1	< **0.001**
		− 195 to − 45 HU	1036	− 88.5	− 1.8	− 1.9	< **0.001**	633	− 92.8	− 1.3	− 1.4	< **0.001**
		− 205 to − 51 HU	1036	− 91.9	− 1.5	− 1.6	< **0.001**	633	− 95.4	− 1.2	− 1.2	< **0.001**
		− 250 to − 50 HU	1036	− 93.9	− 0.9	− 1.0	< **0.001**	633	− 96.7	− 0.8	− 0.8	< **0.001**
	L3	− 150 to − 50 HU	1025	− 90.1	− 1.2	− 1.3	< **0.001**	627	− 94.1	− 0.9	− 0.9	< **0.001**
		− 190 to − 30 HU	1025	− 83.3	− 1.9	− 2.3	< **0.001**	627	− 88.6	− 1.8	− 1.9	< **0.001**
		− 195 to − 45 HU	1025	− 90.4	− 1.5	− 1.6	< **0.001**	627	− 94.1	− 1.4	− 1.5	< **0.001**
		− 205 to − 51 HU	1025	− 93.5	− 1.3	− 1.3	< **0.001**	627	− 96.5	− 1.3	− 1.3	< **0.001**
		− 250 to − 50 HU	1025	− 95.5	− 0.7	− 0.8	< **0.001**	627	− 97.7	− 1.0	− 1.0	< **0.001**
	L4	− 150 to − 50 HU	966	− 90.7	− 1.3	− 1.4	< **0.001**	580	− 92.9	− 1.3	− 1.4	< **0.001**
		− 190 to − 30 HU	966	− 83.8	− 2.2	− 2.5	< **0.001**	580	− 86.9	− 2.1	− 2.4	< **0.001**
		− 195 to − 45 HU	966	− 90.7	− 1.7	− 1.9	< **0.001**	580	− 92.7	− 1.7	− 1.8	< **0.001**
		− 205 to − 51 HU	966	− 93.8	− 1.5	− 1.6	< **0.001**	580	− 95.2	− 1.6	− 1.6	< **0.001**
		− 250 to − 50 HU	966	− 95.4	− 1.1	− 1.1	< **0.001**	580	− 96.2	− 1.2	− 1.2	< **0.001**

*P*-values from paired t-test for T10–L4 with null hypothesis $\mu _d=0$. Negative values indicate contrast value lower than non-contrast value, on average. *P*-values <0.01 in bold.

**Figure 1:**
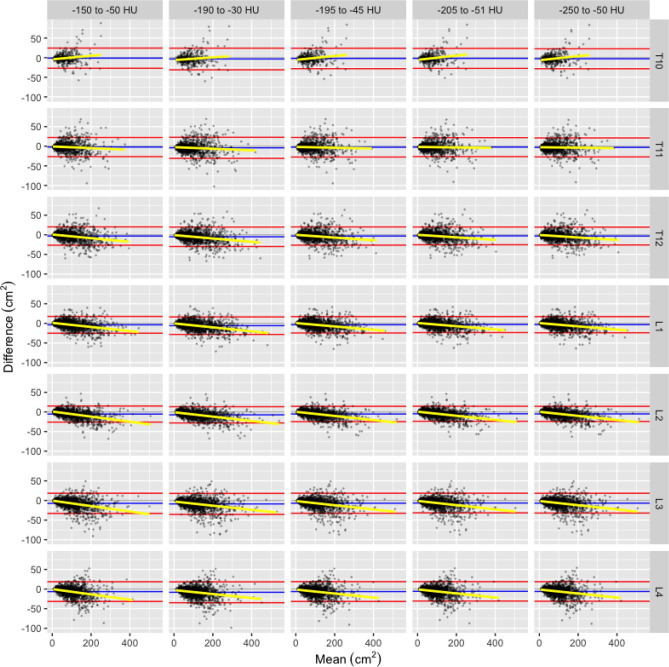
Bland-Altman plot of VFA (cm^2^) by vertebra number, and HU range showing each observation (black point), mean (blue line) and 1.96 standard deviations (red lines) of the paired differences (contrast-non-contrast), linear best-fit regression (yellow line).

**Figure 2:**
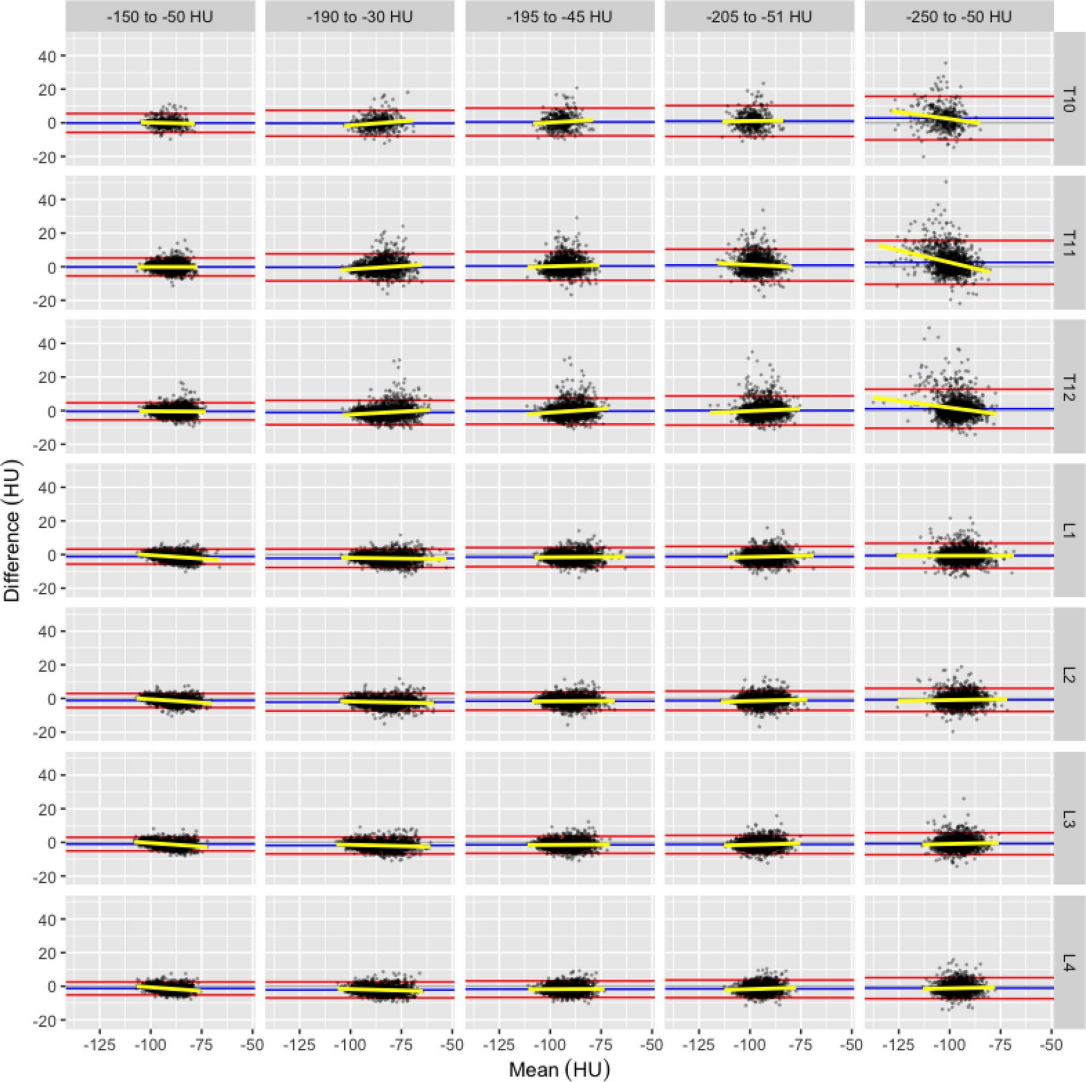
Bland-Altman plot of VFRA (HU) by vertebra number, and HU range showing each observation (black point), mean (blue line) and 1.96 standard deviations (red lines) of the paired differences (contrast-non-contrast), linear best-fit regression (yellow line).

For both VFA and VFRA, Bland-Altman plots of agreement demonstrated proportional bias at all vertebra levels and in all HU ranges; as the mean value increased there was a trend toward greater differences and the variance of the differences increased (Figs. 1 and 2). For VFA, the trend was positive at T10, neutral/negative at T11, and negative for T12 through L4. For VFRA, trends varied by vertebra level and HU range. Comparing HU ranges, the variance of VFA was fairly consistent across ranges, however, the variance of VFRA increased as the HU range increased.

### Fat HU range

Compared to the − 205 to − 50 HU reference range, VFA was lower for the − 150 to − 50 HU range, and higher for all other ranges at all vertebra levels in both sexes and for both contrast and non-contrast scans (Tables 5 and 6). The largest differences were observed with the − 190 to − 30 HU range, which ranged from 15.4% (L3) to 20.2% (T10) in females and from 10.8% (L3) to 14.8% (T10) in males for contrast scans (Table 5), and from 17.1% (L3) to 22.2% (T12) in females and from 11.8% (L3) to 17.0% (T10) in males for non-contrast scans (Table 6). The − 195 to − 45 HU range showed the second largest (positive) differences, followed by the − 150 to − 50 HU (negative), and finally the − 250 to − 50 HU range (positive) in both contrast and non-contrast scans.

**Table 5:** Contrast scan mean VFA paired differences (comparison range$-$reference range) raw ($\mu _d$) and as percent of overall mean reference range value ($\mu _d/\mu _r$).

			Female					Male				
		HU range	n	*μ*_*r*_	*μ*_*d*_	*μ*_*d*_.*μ*_*r*_	*P*	n	*μ*_*r*_	*μ*_*d*_	*μ*_*d*_.*μ*_*r*_	*P*
VFA (cm^2^)	T10	− 150 to − 50 HU	283	52.1	− 2.3	− 4.4	0.000	165	98.2	− 3.9	− 4.0	0.000
		− 190 to − 30 HU	283	52.1	10.5	20.2	0.000	165	98.2	14.5	14.8	0.000
		− 195 to − 45 HU	283	52.1	2.8	5.3	0.000	165	98.2	3.8	3.9	0.000
		− 250 to − 50 HU	283	52.1	1.8	3.5	0.000	165	98.2	2.3	2.4	0.000
	T11	− 150 to − 50 HU	814	51.6	− 1.6	− 3.2	0.000	494	107.6	− 3.5	− 3.2	0.000
		− 190 to − 30 HU	814	51.6	10.3	19.9	0.000	494	107.6	15.1	14.0	0.000
		− 195 to − 45 HU	814	51.6	2.8	5.4	0.000	494	107.6	4.2	3.9	0.000
		− 250 to − 50 HU	814	51.6	1.4	2.7	0.000	494	107.6	2.0	1.8	0.000
	T12	− 150 to − 50 HU	1024	58.6	− 1.3	− 2.3	0.000	625	123.9	− 3.1	− 2.5	0.000
		− 190 to − 30 HU	1024	58.6	11.5	19.6	0.000	625	123.9	16.9	13.6	0.000
		− 195 to − 45 HU	1024	58.6	3.2	5.5	0.000	625	123.9	4.8	3.9	0.000
		− 250 to − 50 HU	1024	58.6	1.2	2.0	0.000	625	123.9	1.8	1.4	0.000
	L1	− 150 to − 50 HU	1040	69.6	− 1.5	− 2.2	0.000	635	140.3	− 3.0	− 2.1	0.000
		− 190 to − 30 HU	1040	69.6	13.0	18.7	0.000	635	140.3	18.3	13.0	0.000
		− 195 to − 45 HU	1040	69.6	3.7	5.3	0.000	635	140.3	5.3	3.8	0.000
		− 250 to − 50 HU	1040	69.6	1.3	1.8	0.000	635	140.3	1.8	1.3	0.000
	L2	− 150 to − 50 HU	1036	74.3	− 1.8	− 2.4	0.000	633	141.4	− 3.2	− 2.3	0.000
		− 190 to − 30 HU	1036	74.3	12.8	17.2	0.000	633	141.4	16.5	11.7	0.000
		− 195 to − 45 HU	1036	74.3	3.6	4.8	0.000	633	141.4	4.8	3.4	0.000
		− 250 to − 50 HU	1036	74.3	1.3	1.8	0.000	633	141.4	1.7	1.2	0.000
	L3	− 150 to − 50 HU	1025	77.0	− 2.0	− 2.6	0.000	627	131.9	− 3.0	− 2.3	0.000
		− 190 to − 30 HU	1025	77.0	11.9	15.4	0.000	627	131.9	14.2	10.8	0.000
		− 195 to − 45 HU	1025	77.0	3.3	4.3	0.000	627	131.9	4.1	3.1	0.000
		− 250 to − 50 HU	1025	77.0	1.3	1.7	0.000	627	131.9	1.5	1.1	0.000
	L4	− 150 to − 50 HU	966	79.8	− 2.0	− 2.5	0.000	580	116.3	− 2.2	− 1.9	0.000
		− 190 to − 30 HU	966	79.8	12.8	16.0	0.000	580	116.3	14.7	12.6	0.000
		− 195 to − 45 HU	966	79.8	3.6	4.5	0.000	580	116.3	4.2	3.6	0.000
		− 250 to − 50 HU	966	79.8	1.3	1.7	0.000	580	116.3	1.4	1.2	0.000

*P*-values from paired t-test for T10-L4 with null hypothesis $\mu _d=0$. Negative values indicate comparison range value lower than reference range value, on average. Reference range is − 205 to − 51 HU.* P*-values < 0.01 in bold.

**Table 6:** Non-contrast scan mean VFA paired differences (comparison range–reference range) raw ($\mu _d$) and as percent of overall mean reference range value ($\mu _r$).

		HU range	Female					Male				
			n	*μ*_*r*_	*μ*_*d*_	*μ*_*d*_.*μ*_*r*_	*P*	n	*μ*_*d*_.*μ*_*r*_	*μ*_*d*_	*μ*_*d*_.*μ*_*r*_	*P*
VFA (cm^2^)	T10	− 150 to − 50 HU	283	55.6	− 3.0	− 5.3	0.000	165	97.1	− 4.3	− 4.4	0.000
		− 190 to − 30 HU	283	55.6	11.9	21.4	0.000	165	97.1	16.5	17.0	0.000
		− 195 to − 45 HU	283	55.6	2.9	5.3	0.000	165	97.1	4.1	4.2	0.000
		− 250 to − 50 HU	283	55.6	2.7	4.9	0.000	165	97.1	3.5	3.6	0.000
	T11	− 150 to − 50 HU	814	53.9	− 2.0	− 3.7	0.000	494	109.6	− 3.3	− 3.0	0.000
		− 190 to − 30 HU	814	53.9	11.9	22.0	0.000	494	109.6	17.3	15.8	0.000
		− 195 to − 45 HU	814	53.9	3.0	5.6	0.000	494	109.6	4.5	4.1	0.000
		− 250 to − 50 HU	814	53.9	2.1	3.8	0.000	494	109.6	2.8	2.6	0.000
	T12	− 150 to − 50 HU	1024	60.7	− 1.2	− 2.0	0.000	625	127.6	− 2.2	− 1.8	0.000
		− 190 to − 30 HU	1024	60.7	13.5	22.2	0.000	625	127.6	19.7	15.4	0.000
		− 195 to − 45 HU	1024	60.7	3.6	5.9	0.000	625	127.6	5.4	4.2	0.000
		− 250 to − 50 HU	1024	60.7	1.6	2.6	0.000	625	127.6	2.3	1.8	0.000
	L1	− 150 to − 50 HU	1040	71.7	− 1.1	− 1.5	0.000	635	145.0	− 1.9	− 1.3	0.000
		− 190 to − 30 HU	1040	71.7	15.6	21.8	0.000	635	145.0	21.5	14.8	0.000
		− 195 to − 45 HU	1040	71.7	4.2	5.9	0.000	635	145.0	6.0	4.1	0.000
		− 250 to − 50 HU	1040	71.7	1.6	2.2	0.000	635	145.0	2.2	1.5	0.000
	L2	− 150 to − 50 HU	1036	77.9	− 1.3	− 1.7	0.000	633	148.0	− 2.1	− 1.4	0.000
		− 190 to − 30 HU	1036	77.9	15.3	19.6	0.000	633	148.0	19.4	13.1	0.000
		− 195 to − 45 HU	1036	77.9	4.2	5.3	0.000	633	148.0	5.4	3.7	0.000
		− 250 to − 50 HU	1036	77.9	1.7	2.1	0.000	633	148.0	2.1	1.4	0.000
	L3	− 150 to − 50 HU	1025	81.9	− 1.4	− 1.7	0.000	627	141.1	− 1.9	− 1.3	0.000
		− 190 to − 30 HU	1025	81.9	14.0	17.1	0.000	627	141.1	16.7	11.8	0.000
		− 195 to − 45 HU	1025	81.9	3.8	4.7	0.000	627	141.1	4.7	3.3	0.000
		− 250 to − 50 HU	1025	81.9	1.6	2.0	0.000	627	141.1	1.8	1.3	0.000
	L4	− 150 to − 50 HU	966	84.9	− 1.4	− 1.6	0.000	580	123.5	− 1.5	− 1.2	0.000
		− 190 to − 30 HU	966	84.9	15.1	17.8	0.000	580	123.5	17.3	14.0	0.000
		− 195 to − 45 HU	966	84.9	4.2	4.9	0.000	580	123.5	4.8	3.9	0.000
		− 250 to − 50 HU	966	84.9	1.6	1.9	0.000	580	123.5	1.8	1.4	0.000

*P*-values from paired t-test for T10–L4 with null hypothesis $\mu _d=0$. Negative values indicate comparison range value lower than reference range value, on average. Reference range is − 205 to − 51 HU.* P*-values < 0.01 in bold.

Compared to the − 205 to − 50 HU reference range, VFRA was lower for the − 250 to − 50 HU range, and higher for all other ranges at all vertebra levels in both sexes and for both contrast and non-contrast scans (Tables 7 and 8). The largest differences were observed with the − 190 to − 30 HU range, which ranged from 9.8% (L4) to 12.6% (T10) in females and from 7.6% (L3) to 10.1% (T10) in males for contrast scans (Table 7), and from 10.6% (L4) to 13.6% (T10, T11) in females and from 8.2% (L3) to 11.7% (T10) in males for non-contrast scans (Table 8). The − 150 to − 50 HU range showed the second largest (positive) differences, followed by the − 195 to − 45 HU range (positive), and finally the − 250 to − 50 HU range (negative) in both contrast and non-contrast scans.

**Table 7:** Contrast scan mean VFRA paired differences (comparison range–reference range) raw ($\mu _d$) and as percent of absolute overall mean reference range value ($\mu _d/|\mu _r|$).

			Female					Male				
		HU range	n	$\mu _r$	$\mu _d$	$\mu _d/|\mu _r|$ (%)	*P*	n	$\mu _r$	$\mu _d$	$\mu _d/|\mu _r|$ (%)	*P*
VFRA (*HU*)	T10	− 150 to − 50 HU	283	− 97.0	6.2	6.3	0.000	165	− 99.9	5.2	5.2	0.000
		− 190 to − 30 HU	283	− 97.0	12.2	12.6	0.000	165	− 99.9	10.1	10.1	0.000
		− 195 to − 45 HU	283	− 97.0	4.0	4.1	0.000	165	− 99.9	3.2	3.2	0.000
		− 250 to − 50 HU	283	− 97.0	− 4.0	− 4.1	0.000	165	− 99.9	− 2.8	− 2.8	0.000
	T11	− 150 to − 50 HU	814	− 94.7	5.0	5.2	0.000	494	− 98.3	4.4	4.5	0.000
		− 190 to − 30 HU	814	− 94.7	11.6	12.3	0.000	494	− 98.3	9.6	9.7	0.000
		− 195 to − 45 HU	814	− 94.7	3.7	3.9	0.000	494	− 98.3	3.0	3.0	0.000
		− 250 to − 50 HU	814	− 94.7	− 3.0	− 3.1	0.000	494	− 98.3	− 2.1	− 2.1	0.000
	T12	− 150 to − 50 HU	1024	− 92.4	3.8	4.1	0.000	625	− 96.2	3.5	3.7	0.000
		− 190 to − 30 HU	1024	− 92.4	11.1	12.0	0.000	625	− 96.2	9.0	9.4	0.000
		− 195 to − 45 HU	1024	− 92.4	3.5	3.7	0.000	625	− 96.2	2.8	2.9	0.000
		− 250 to − 50 HU	1024	− 92.4	− 1.8	− 2.0	0.000	625	− 96.2	− 1.3	− 1.3	0.000
	L1	− 150 to − 50 HU	1040	− 92.2	3.4	3.7	0.000	635	− 95.5	3.0	3.1	0.000
		− 190 to − 30 HU	1040	− 92.2	10.5	11.4	0.000	635	− 95.5	8.4	8.8	0.000
		− 195 to − 45 HU	1040	− 92.2	3.3	3.6	0.000	635	− 95.5	2.6	2.7	0.000
		− 250 to − 50 HU	1040	− 92.2	− 1.4	− 1.5	0.000	635	− 95.5	− 0.9	− 0.9	0.000
	L2	− 150 to − 50 HU	1036	− 93.4	3.5	3.7	0.000	633	− 96.6	2.9	3.0	0.000
		− 190 to − 30 HU	1036	− 93.4	10.1	10.8	0.000	633	− 96.6	7.9	8.2	0.000
		− 195 to − 45 HU	1036	− 93.4	3.1	3.4	0.000	633	− 96.6	2.4	2.5	0.000
		− 250 to − 50 HU	1036	− 93.4	− 1.4	− 1.5	0.000	633	− 96.6	− 0.9	− 0.9	0.000
	L3	− 150 to − 50 HU	1025	− 94.8	3.5	3.7	0.000	627	− 97.8	2.8	2.9	0.000
		− 190 to − 30 HU	1025	− 94.8	9.5	10.0	0.000	627	− 97.8	7.5	7.6	0.000
		− 195 to − 45 HU	1025	− 94.8	3.0	3.1	0.000	627	− 97.8	2.3	2.3	0.000
		− 250 to − 50 HU	1025	− 94.8	− 1.4	− 1.5	0.000	627	− 97.8	− 0.8	− 0.9	0.000
	L4	− 150 to − 50 HU	966	− 95.3	3.3	3.4	0.000	580	− 96.7	2.5	2.6	0.000
		− 190 to − 30 HU	966	− 95.3	9.3	9.8	0.000	580	− 96.7	7.7	8.0	0.000
		− 195 to − 45 HU	966	− 95.3	2.9	3.0	0.000	580	− 96.7	2.3	2.4	0.000
		− 250 to − 50 HU	966	− 95.3	− 1.2	− 1.2	0.000	580	− 96.7	− 0.7	− 0.7	0.000

*P*− values from paired t-test for T10–L4 with null hypothesis $\mu _d=0$. Negative values indicate comparison range value lower than reference range value, on average. Reference range is − 205 to − 51 HU. *P*-values < 0.01 in bold.

**Table 8:** Non-contrast scan mean VFRA paired differences (comparison range–reference range) raw ($\mu _d$) and as percent of absolute overall mean reference range value ($\mu _d/|\mu _r|$).

			Female					Male				
		HU range	n	$\mu _r$	$\mu _d$	$\mu _d/|\mu _r|$ (%)	*P*	n	$\mu _r$	$\mu _d$	$\mu _d/|\mu _r|$ (%)	*P*
VFRA (*HU*)	T10	− 150 to − 50 HU	283	− 98.1	7.4	7.5	0.000	165	− 101.0	6.3	6.3	0.000
		− 190 to − 30 HU	283	− 98.1	13.3	13.6	0.000	165	− 101.0	11.8	11.7	0.000
		− 195 to − 45 HU	283	− 98.1	4.5	4.5	0.000	165	− 101.0	3.9	3.8	0.000
		− 250 to − 50 HU	283	− 98.1	− 5.7	− 5.9	0.000	165	− 101.0	− 4.6	− 4.5	0.000
	T11	− 150 to − 50 HU	814	− 96.0	6.4	6.6	0.000	494	− 98.8	5.0	5.0	0.000
		− 190 to − 30 HU	814	− 96.0	13.1	13.6	0.000	494	− 98.8	10.7	10.9	0.000
		− 195 to − 45 HU	814	− 96.0	4.3	4.5	0.000	494	− 98.8	3.4	3.5	0.000
		− 250 to − 50 HU	814	− 96.0	− 4.8	− 5.0	0.000	494	− 98.8	− 3.4	− 3.5	0.000
	T12	− 150 to − 50 HU	1024	− 92.8	4.6	5.0	0.000	625	− 95.9	3.6	3.7	0.000
		− 190 to − 30 HU	1024	− 92.8	12.5	13.4	0.000	625	− 95.9	9.9	10.4	0.000
		− 195 to − 45 HU	1024	− 92.8	3.9	4.2	0.000	625	− 95.9	3.1	3.2	0.000
		− 250 to − 50 HU	1024	− 92.8	− 3.1	− 3.3	0.000	625	− 95.9	− 2.1	− 2.2	0.000
	L1	− 150 to − 50 HU	1040	− 90.8	3.5	3.8	0.000	635	− 94.3	2.7	2.8	0.000
		− 190 to − 30 HU	1040	− 90.8	11.6	12.8	0.000	635	− 94.3	9.1	9.7	0.000
		− 195 to − 45 HU	1040	− 90.8	3.6	3.9	0.000	635	− 94.3	2.8	3.0	0.000
		− 250 to − 50 HU	1040	− 90.8	− 2.0	− 2.2	0.000	635	− 94.3	− 1.4	− 1.4	0.000
	L2	− 150 to − 50 HU	1036	− 91.9	3.5	3.8	0.000	633	− 95.4	2.6	2.7	0.000
		− 190 to − 30 HU	1036	− 91.9	11.0	12.0	0.000	633	− 95.4	8.6	9.0	0.000
		− 195 to − 45 HU	1036	− 91.9	3.4	3.7	0.000	633	− 95.4	2.6	2.7	0.000
		− 250 to − 50 HU	1036	− 91.9	− 2.0	− 2.1	0.000	633	− 95.4	− 1.3	− 1.3	0.000
	L3	− 150 to − 50 HU	1025	− 93.5	3.4	3.6	0.000	627	− 96.5	2.4	2.5	0.000
		− 190 to − 30 HU	1025	− 93.5	10.2	10.9	0.000	627	− 96.5	7.9	8.2	0.000
		− 195 to − 45 HU	1025	− 93.5	3.2	3.4	0.000	627	− 96.5	2.4	2.5	0.000
		− 250 to − 50 HU	1025	− 93.5	− 1.9	− 2.1	0.000	627	− 96.5	− 1.2	− 1.2	0.000
	L4	− 150 to − 50 HU	966	− 93.8	3.1	3.3	0.000	580	− 95.2	2.3	2.4	0.000
		− 190 to − 30 HU	966	− 93.8	10.0	10.6	0.000	580	− 95.2	8.3	8.7	0.000
		− 195 to − 45 HU	966	− 93.8	3.1	3.3	0.000	580	− 95.2	2.5	2.6	0.000
		− 250 to − 50 HU	966	− 93.8	− 1.6	− 1.7	0.000	580	− 95.2	− 1.1	− 1.1	0.000

*P*-values from paired t-test for T10–L4 with null hypothesis $\mu _d=0$. Negative values indicate comparison range value lower than reference range value, on average. Reference range is − 205 to − 51 HU. *P*-values < 0.01 in bold.

### Outlier investigation

**Figure 3:**
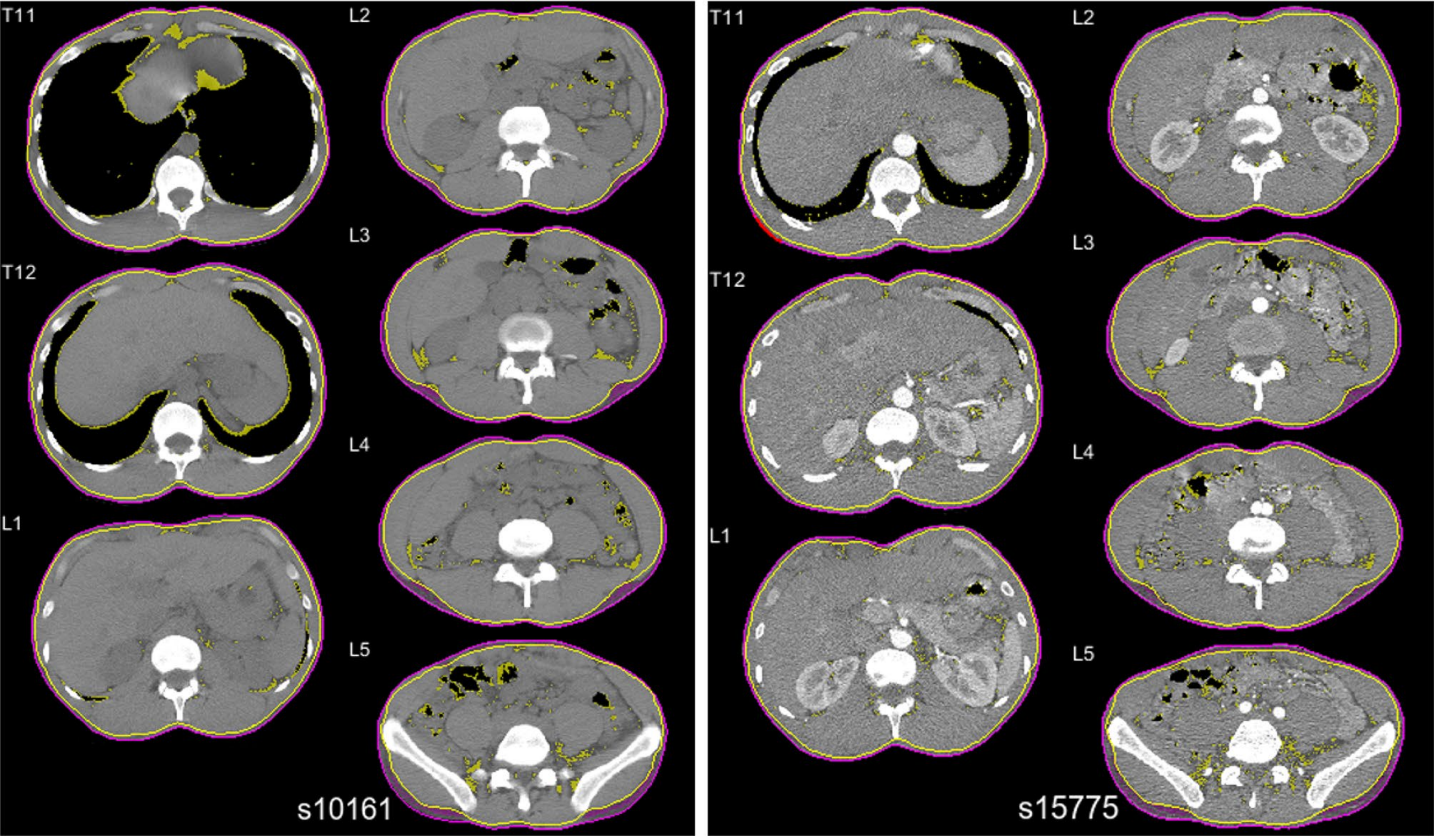
Example of healthy 36 y/o male non-contrast (left) and contrast (right) CT axial slices, showing T11–L5 visceral fat area (yellow-shaded region), subcutaneous fat area (purple shaded region), outer abdominal fascia boundary (yellow line), and skin boundary (purple line). Images demonstrate different lung inflation between contrast and non-contrast scans and resulting shift in organ and fat pixels.

Individuals with paired differences greater than 4 s.d. above/below the mean difference were identified and investigated individually as possible outliers. These pairs displayed distinct differences in lung inflation, resulting in vertical shift of organ locations and large differences in axial visceral non-muscular contents between the two phases of scan. Differences were more visually apparent at the thoracic levels where lung air was directly observable, and less apparent in the lumbar region where differences were due to more subtle shifts in major organs including liver, kidney, spleen, and bowel (Fig. 3).

## Discussion

Both the presence of intravenous contrast and the particular HU range used to define visceral fat pixels affected measurements of visceral fat cross-sectional area and mean radiation attenuation on CT.

Analysis of contrast versus non-contrast scans showed that VFA was higher in non-contrast scans compared to contrast scans, whereas VFRA was lower in contrast scans compared to non-contrast scans. The difference in HU was in general quite close to zero, however, Bland-Altman plots demonstrated proportional bias-differences increasing with the mean value-particularly in VFA measurements. The differences due to contrast increased as the area or density of visceral fat increased. Thus, patients with greater visceral fat or greater fat density had greater measurement differences between scans with and without contrast. Therefore, to fairly compare fat measurements for patients with low and high visceral fat, contrast and non-contrast scans should not be mixed within the same study.

Additionally, measurement differences due to HU range were much greater than those due to contrast. In particular, the HU range of − 190 to − 30 showed the greatest differences in both VFA and VFRA. This range was the only range that includes HU values greater than − 45 HU, suggesting that the large difference was primarily due to pixels between − 45 and − 30 HU being counted as fat tissue. Hence, we conclude that visceral fat measurements derived using different fat HU ranges cannot be directly compared.

Outlier investigation showed that reliability of both VFA and VFRA measurements are strongly dependent upon breath cycle; individuals whose paired scans demonstrated large differences in lung inflation also demonstrated large differences in VFA and/or VFRA, particularly in the thoracic region. Caution should be exercised when using visceral fat measurements from the thoracic region as these measurements will be strongly affected by the amount of air held in the lungs at the time of the scan.

This study has important limitations. Our cohort may not be nationally or globally representative, though it is not specific to a particular race or ethnicity. These reference values have not been tested against clinical outcomes and were computed from kidney donor CT protocols only; we did not evaluate the effect of different kVp, mA, convolution kernel, contrast dosage, and/or slice thicknesses used in other protocols nor did we evaluate the effect of various disease states, height, or BMI on these measurements. Because we used retrospective scans, we could not control the slice thickness used in each scan and slice thickness was correlated with the type of scan due to scan protocols used. The vast majority of non-contrast scans used 5mm slice thickness whereas the majority of contrast scans used 2.5mm or smaller slice thickness, resulting in ’sharper’ contrast images and ’blurrier’ non-contrast images. The effect of this difference was not specifically addressed in this manuscript, though visual inspection of distributions showed no large, obvious deviations.

Clinical CT scans obtained in the normal course of patient care can potentially be used for detailed cross-sectional visceral fat evaluation. However, scans may not include vertebral levels for which reference values have been defined (e.g., L3) and may or may not use contrast enhancement. Furthermore, we have shown that the fat HU range used to identify fat pixels can significantly change the resulting measurements, rendering it perhaps one of the most important choices made in CT fat measurement. We report healthy population reference values for a wide range of vertebral levels, in both contrast and non-contrast phases, and using multiple different fat HU ranges. These values provide strong evidence towards standardizing visceral fat area and radiation attenuation measurements and provide a healthy reference population for other studies to compare against.

## Methods

### Study cohort

We retrospectively studied persons who underwent CT scans at Michigan Medicine as part of evaluation for kidney donation between 1999 and 2017. We have previously studied a subset of these kidney donor candidates as a healthy reference population for skeletal muscle^25^.

Patient age, sex, height, and weight were obtained from their medical record proximal to the date of evaluation for kidney donation, and the month and year of the evaluation appointment was recorded^27^. Candidates were included if they had a CT scan performed as part of evaluation for kidney donation, were deemed healthy enough to donate, had age, sex, height, and weight recorded in their electronic medical record, had both contrast-enhanced and non-contrast-enhanced series available, and had a fascia boundary that was fully visible in the display field of view.

Body mass index (BMI) was computed and categorized into groups according the World Health Organization International Classification standard^28^. Race, unavailable for 25% of the cohort, was reported but not specifically analyzed.

CT imaging was extracted for 2,902 total donor candidates between the ages of 18 and 75. The *n* = 1,677 candidates of all ages having both a contrast and non-contrast series scan were used in this analysis.

All candidates included in the analysis were scanned using the GE ‘Standard’ reconstruction algorithm (which is optimized for visualizing soft tissue) at 120 kVp in a Discovery or LightSpeed scanner. Non-contrast scans used 2.5 (2007–2010), 3.75 (2008), or 5 mm (1999–2017) slice thickness and contrast scans used 0.625 (2005–2010), 1.25 (2002–2008), 2.5 (1999–2002, 2010–2017), 3.75 (1999), and 5 mm (2002–2016) slice thickness. Tube current was automatically modulated in proportion to body mass.

### CT image processing

**Figure 4:**
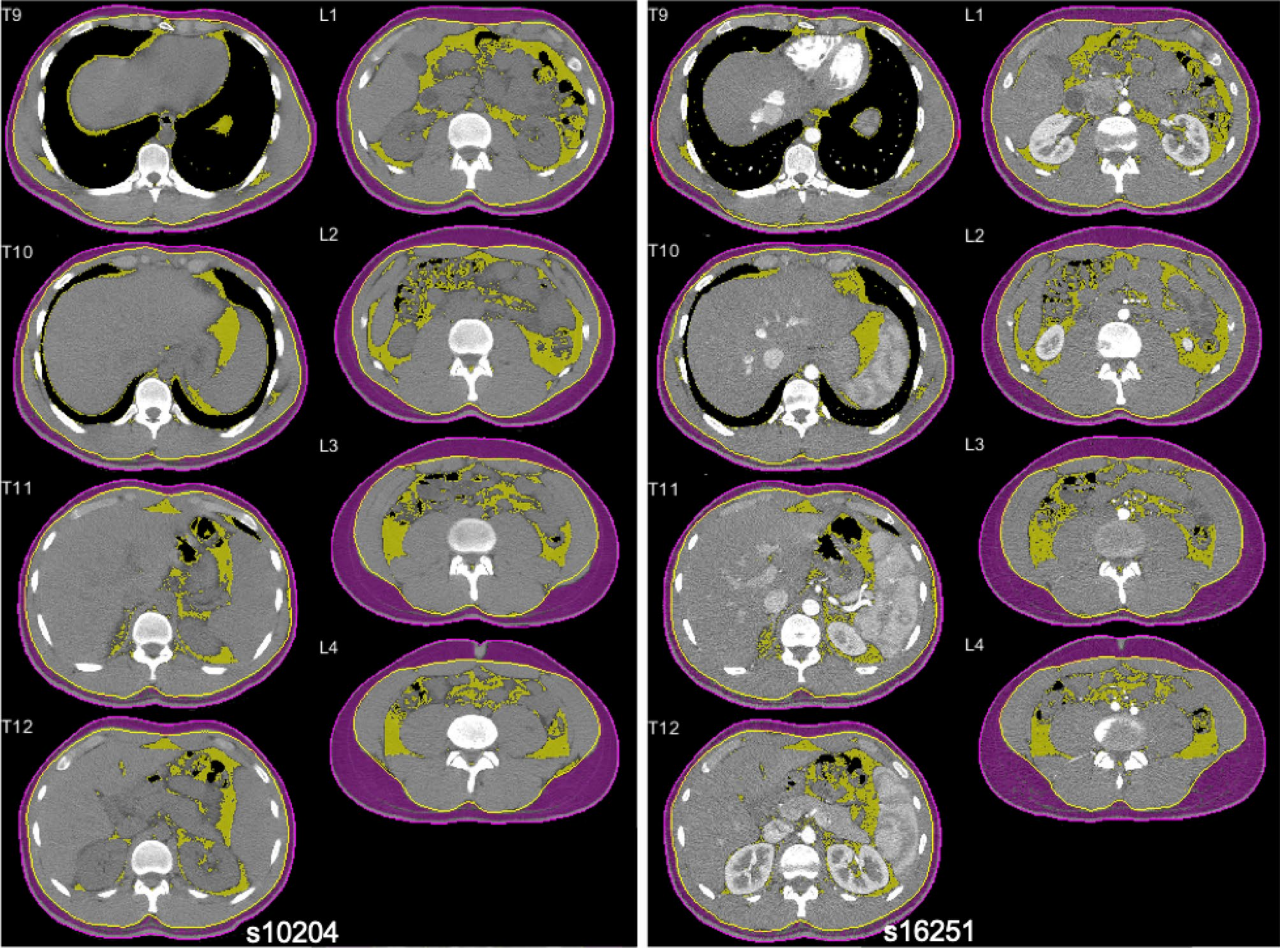
Example of healthy 20 y/o male non-contrast (left) and contrast (right) CT axial slices, showing T10-L4 visceral fat area (yellow-shaded region), subcutaneous fat area (purple shaded region), outer abdominal fascia boundary (yellow line), and skin boundary (purple line). Portions of skin boundary that are coincident with the scan field of view are highlighted in red.

After being transferred into a spatial database, CT images were processed using Analytic Morphomics, a semi-automated image analysis method that has been previously described^24, 29, 30^. A combination of automated and user-guided algorithms written in Matlab (The Mathworks Inc, Natick, MA) identified the vertebral bodies to serve as an anatomical coordinate reference system. Next, the outer abdominal fascia and skin boundary were identified at all available vertebral levels to create enclosed regions of interest, which were confirmed by multiple trained researchers (Fig. 4).

Measurements were taken at each vertebral level between T10 through L4. Sample size at each vertebra level varied due to differences in anatomy included in each subject’s scan. Vertebra levels that did not include both a non-contrast and contrast measurement were excluded.

Fat measurements were computed using the axial slice nearest the inferior aspect of each vertebral body. Visceral fat area (VFA) was computed as the total area of pixels enclosed by the outer abdominal fascia, falling within five different fat Hounsfield Unit (HU) ranges: (1) − 205 to − 51 HU (our reference), (2) − 150 to − 50, (3) − 195 to − 45, (4) − 190 to − 30, (5) − 250 to − 50 HU. Mean radiation attenuation (VFRA) was computed as the mean HU of all pixels included in VFA.

### Statistical methods

Male and female demographics, CT parameters, and fat measurements are shown separately as mean +/− s.d. for continuous variables and proportion for categorical variables. Continuous means were compared using two-tailed t-tests assuming unequal variance and categorical proportions were compared using the Chi-squared test.

The within-subject difference between (1) enhanced and non-enhanced fat measures, and (2) between each HU range and the reference range were assessed using paired t-tests. The mean difference and the mean difference expressed as a percentage of the (1) enhanced and (2) reference measurement are reported for each test. Fat HU ranges were analyzed separately in enhanced and non-enhanced scans.

Bland-Altman plots were used to visualize the agreement between pairs of measurements^31^.

The sex-specific mean and standard deviation of each fat measure were calculated independently for vertebral levels from T10 to L4.

An alpha level of .01 was used to determine statistical significance. All statistical tests were performed in R version 4.0.2^32^, using the package ‘ggplot2’^33^ for data visualization.

### Ethical approval and informed consent

This study was approved by the Institutional Review Board of Michigan Medicine. All methods were performed in accordance with the relevant guidelines and regulations of the United States. Because existing CT scans were used retrospectively, the requirement for informed consent was waived by the Institutional Review Board of the University of Michigan.

## Data Availability

The datasets generated during and/or analyzed during the current study are available from the corresponding author upon reasonable request.
